# Case Report: Marked intrafamilial variability and recurrent fever-associated alopecia in *SREBF1*-associated hereditary mucoepithelial dysplasia

**DOI:** 10.3389/fmed.2026.1910789

**Published:** 2026-10-01

**Authors:** Mingyuan Xu, Lei Wang, Yonglong Xu, Dingquan Yang

**Affiliations:** 1 Beijing University of Chinese Medicine, Beijing, China; 2 Department of Dermatology, China-Japan Friendship Hospital, National Center for Integrative Medicine, Beijing, China

**Keywords:** case report, fever-associated alopecia, hereditary mucoepithelial dysplasia, intrafamilial variability, *SREBF1*

## Abstract

*SREBF1*-associated hereditary mucoepithelial dysplasia (HMD) is a rare autosomal dominant disorder involving the hair, skin, mucosa, and eyes. We report a 5-year-old boy with recurrent non-scarring alopecia beginning at 18 months of age, together with blepharokeratoconjunctivitis, dry eye, corneal ulceration, recurrent oral ulcers, and nail pitting. Hair loss repeatedly worsened during or shortly after febrile episodes, most prominently in the occipital scalp, and was followed by gradual regrowth. Clinical exome sequencing identified a heterozygous *SREBF1* NM_004176.5:c.1580G>A (p.Arg527His) variant. His father carried the same variant but had a much milder phenotype, with childhood-onset occipital and temporal alopecia and mild ocular symptoms. The variant was not detected in his clinically unaffected sister. Hair regrowth was observed after topical scalp treatment, although occipital shedding recurred during follow-up. This case adds further evidence of marked intrafamilial variability in *SREBF1*-associated HMD and records a recurrent pattern of hair loss temporally associated with fever.

## 1 Introduction

Hereditary mucoepithelial dysplasia (HMD) is a rare autosomal dominant disorder affecting the hair, skin, mucosa, and eyes. Heterozygous *SREBF1* variants have been reported in both HMD and autosomal dominant ichthyosis follicularis, atrichia and photophobia (AD-IFAP), and these conditions are now considered part of a continuous *SREBF1*-related phenotypic spectrum (1–3). This spectrum is distinct from classic X-linked *MBTPS2*-related IFAP.

We report a father and son carrying the *SREBF1* c.1580G>A (p.Arg527His) variant. The child had early-onset recurrent alopecia, severe ocular surface inflammation, and fever-associated worsening of hair loss, while the father showed only mild alopecia and ocular symptoms. The case illustrates marked intrafamilial variability and the potential for *SREBF1*-associated HMD to mimic immune-mediated disease.

## 2 Case description

A boy aged 5 years and 6 months presented on 2 January 2026 with recurrent hair loss. Hair loss had first developed at 18 months of age, initially involving the frontal and vertex scalp and later extending to the bilateral temporal and occipital regions. The course was fluctuating. According to the parents, worsening hair loss usually occurred on the day of fever or within several days to 1–2 weeks after defervescence, most prominently in the occipital region, followed by gradual regrowth over the subsequent weeks. Similar episodes had occurred repeatedly. During periods of hair loss, 5% topical minoxidil had been used. The eyebrows and eyelashes remained unaffected throughout the course. At presentation on 2 January 2026, approximately 80% of the scalp was involved, with scattered follicular papules and recurrent oral ulcers. Photophobia was not evident.

In February 2024, the patient had been hospitalized for hair loss accompanied by bilateral conjunctival hyperemia. Alopecia areata, immune-mediated alopecia, and connective tissue disease were considered, although no definite autoimmune disease was established. Hydroxychloroquine was subsequently prescribed, and baricitinib was added because immune-mediated alopecia remained a concern. Blepharoconjunctivitis, keratitis, and dry eye had also been documented during the same period. According to the parents, baricitinib was taken for approximately 6 months after the 2024 evaluation and then discontinued; it was not restarted. They reported no appreciable reduction in recurrent shedding during that course. Hydroxychloroquine has continued through the latest history update. The exact baricitinib initiation and cessation dates were not available.

On examination, diffuse hair thinning involved the frontal, vertex, bilateral temporal, and occipital scalp. The remaining hairs were fine and hypopigmented, with broken hairs and exclamation-mark hairs. Scattered follicular papules and eczematous patches were present on the scalp, with similar lesions on both lower legs. Two shallow oral ulcers were present, and mild pitting affected both thumbnails. Baseline clinical photographs are shown in Figure 1.

**Figure 1:**
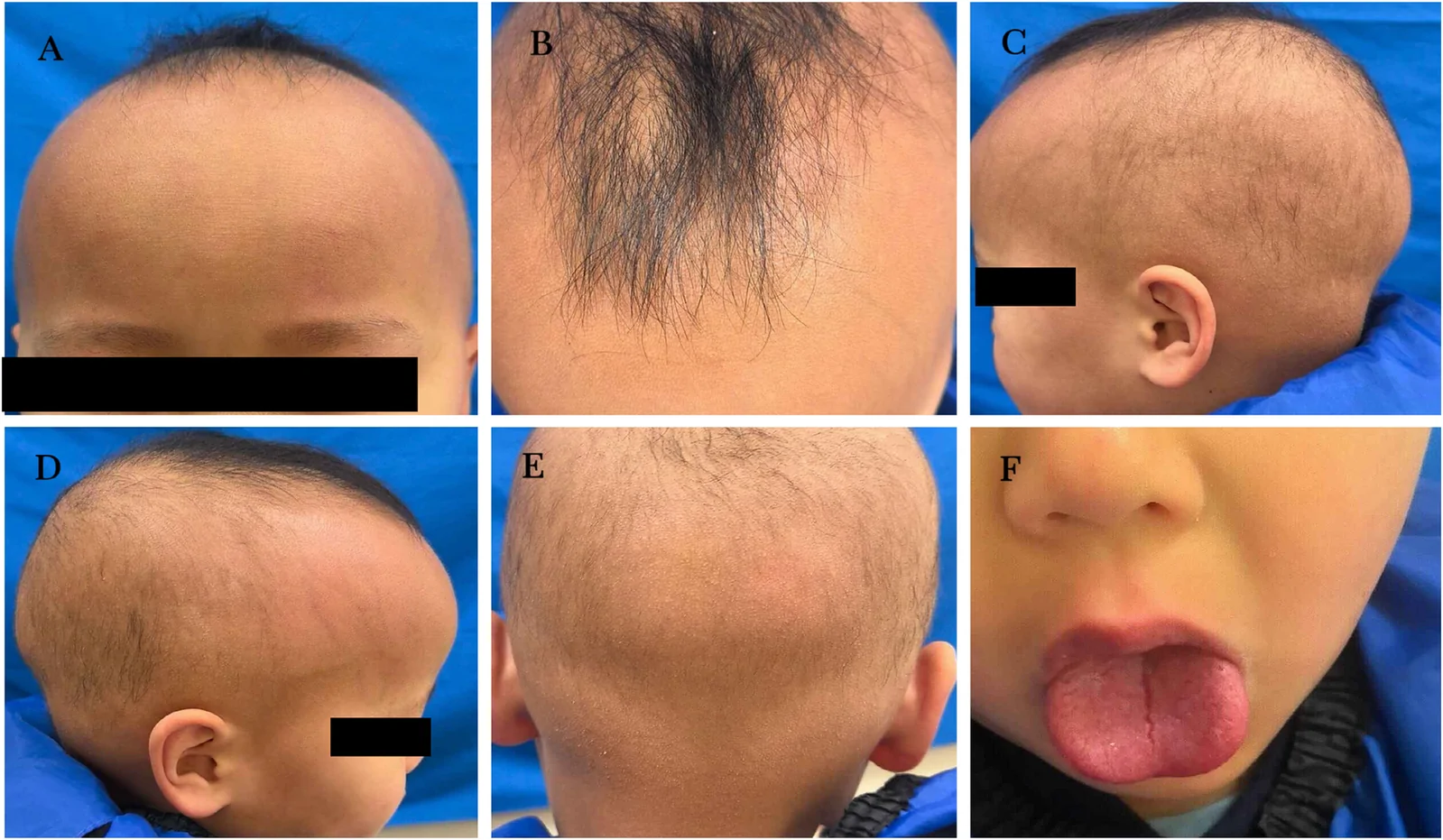
Clinical photographs of the patient at baseline. **(A)** Frontal view. **(B)** Vertex and frontal regions. **(C)** Left lateral view. **(D)** Right lateral view. **(E)** Occipital region. **(F)** Tongue mucosa. Severe diffuse hair thinning is evident across all scalp regions.

The patient's father had developed alopecia at approximately 8–9 years of age, mainly involving the occipital and temporal scalp; his current hair loss was mild. He had a history of recurrent conjunctival hyperemia and dry eye, without obvious photophobia or a known history of corneal ulceration, and had never undergone formal ophthalmologic evaluation. Pitting of the fingernails and toenails had previously been documented. He reported no recurrent oral ulcers, obvious skin roughness, or hyperkeratosis. Neither the patient nor his father had dysphagia, food impaction, recurrent vomiting, or other significant gastrointestinal symptoms. The mother and elder sister were clinically unaffected, and the paternal grandfather had no reported history of alopecia.

The clinical course is summarized in Table 1.

**Table 1:** Clinical timeline of the patient.

Date or age	Clinical course and assessment	Treatment and outcome
Age 18 months	Onset of hair loss involving the frontal and vertex scalp, later extending to temporal and occipital regions.	A fluctuating course with recurrent shedding and gradual regrowth was subsequently reported.
February 2024 Age 3 years 7–8 months	Hospitalization for hair loss and bilateral conjunctival hyperemia. Alopecia areata, immune-mediated alopecia, and connective tissue disease were considered. Blepharoconjunctivitis, keratitis, and dry eye were documented.	Hydroxychloroquine was subsequently prescribed (75 mg/day in the treatment record) and continued. Baricitinib was used later for approximately 6 months, without appreciable improvement according to the parents, then stopped; exact dates were unavailable.
April 2024 Age 3 years 9 months	Clinical exome sequencing identified heterozygous *SREBF1* NM_004176.5:c.1580G>A (p.Arg527His). The same variant was confirmed in the father by Sanger sequencing and was not detected in the elder sister.	The molecular findings supported *SREBF1*-associated HMD.
2 January 2026 Age 5 years 6 months	Dermatology presentation at age 5 years 6 months; approximately 80% of the scalp was affected. Ocular, oral, and nail involvement was present.	Baricitinib had already been stopped after an approximately 6-month course and was not restarted. Hydroxychloroquine continued. A 1:1 mixture of 1 mL 0.1% halcinonide and 1 mL 5% minoxidil was applied twice daily.
6 April 2026 Age 5 years 9 months	Three-month follow-up: the area of scalp alopecia decreased to approximately 20%; trichoscopy and thumbnail pitting improved.	Hair regrowth was observed during treatment; no new recurrence of ocular inflammation was documented.
12 June 2026 Age 5 years 11 months	Five-month follow-up: frontal improvement was largely maintained; marked occipital hair loss recurred.	Treatment and follow-up continued.
27 August 2026 Age 6 years 1 month	Approximately 8-month follow-up: the mother reported recurrent hair loss after fever in July, most prominent occipitally, with gradual regrowth from early August.	Topical scalp treatment continued. No new ocular inflammatory recurrence was documented. At the latest parental update, the mixture and hydroxychloroquine remained in use, with good reported adherence and no reported adverse events.

HMD, hereditary mucoepithelial dysplasia.

## 3 Diagnostic assessment

Routine complete blood count, serum biochemistry, urinalysis, and thyroid function tests showed no significant abnormalities. Immunologic testing showed an antinuclear antibody (ANA) titer of 1:160, weakly positive anti-double-stranded DNA antibodies, and an anti-*β*2-glycoprotein I antibody level of 42.9 RU/mL (reference value <20 RU/mL). Imaging showed multiple enlarged lymph nodes in the cervical, axillary, and inguinal regions. These findings made autoimmune disease an important differential diagnosis at the time; however, when considered together with the clinical presentation and subsequent follow-up, they were insufficient to support a definite diagnosis of connective tissue disease.

Trichoscopy ( × 40) showed yellow dots, black dots, broken hairs, vellus hairs, perifollicular keratotic plugs, and perifollicular pigmentation. Ophthalmologic examination showed mild bilateral lid-margin hyperemia, obstruction of the meibomian gland orifices, and lipid plugging. A central geographic corneal ulcer with stromal haze was present in the right eye. The ophthalmologic diagnoses were bilateral blepharokeratoconjunctivitis (BKC), dry eye, and a right corneal ulcer.

The genetic report, updated on 17 April 2024 following paternal testing, documented clinical exome sequencing. Target coding regions were enriched using a probe-capture method and subjected to high-throughput sequencing. The report described Illumina HiSeq sequencing, with mean target depth >90× and 96% of target regions covered at >20× in its clinical exome methods. Sequence data were processed using Sentieon and aligned to the hg19 reference genome. A heterozygous *SREBF1* variant, NM_004176.5:c.1580G>A (p.Arg527His), corresponding to the genomic variant NC_000017.10:g.17720596C>T, was identified. Because *SREBF1* is located on the negative strand, the direction of the genomic nucleotide change is opposite to that described at the transcript level. Sanger sequencing confirmed the same heterozygous variant in the father, whereas the variant was not detected in the elder sister. The original genetic report recorded an allele count of 6 and a homozygote count of 0 in gnomAD and classified the variant as a disease-causing mutation (DM) in the Human Gene Mutation Database (HGMD).

## 4 Therapeutic intervention and follow-up

At the dermatology assessment in January 2026, baricitinib had already been discontinued. Hydroxychloroquine was continued at the existing regimen (75 mg/day in the treatment record). Topical scalp treatment consisted of 1 mL of 0.1% halcinonide solution mixed with 1 mL of 5% minoxidil solution in a 1:1 volume ratio, with the resulting 2 mL mixture applied twice daily. Thus, the nominal concentrations after mixing were 0.05% halcinonide and 2.5% minoxidil. A compound clobetasol propionate ointment was also used. Ophthalmologic treatment included levofloxacin eye drops and a bandage contact lens to protect the ocular surface. The available history did not specify the baricitinib dose or the detailed dosing schedules of these additional topical and ophthalmic treatments.

At the 3-month follow-up on 6 April 2026, the area of scalp alopecia had decreased from approximately 80% at baseline to approximately 20%, with clear hair regrowth (Figure 2). Trichoscopy showed fewer yellow dots, increased numbers of vellus and terminal hairs, and reduced perifollicular keratosis. Pitting of both thumbnails had also improved (Figure 3). At the 5-month follow-up on 12 June 2026, the improvement in the frontal scalp was largely maintained, but marked hair loss had recurred in the occipital region (Figure 2).

**Figure 2:**
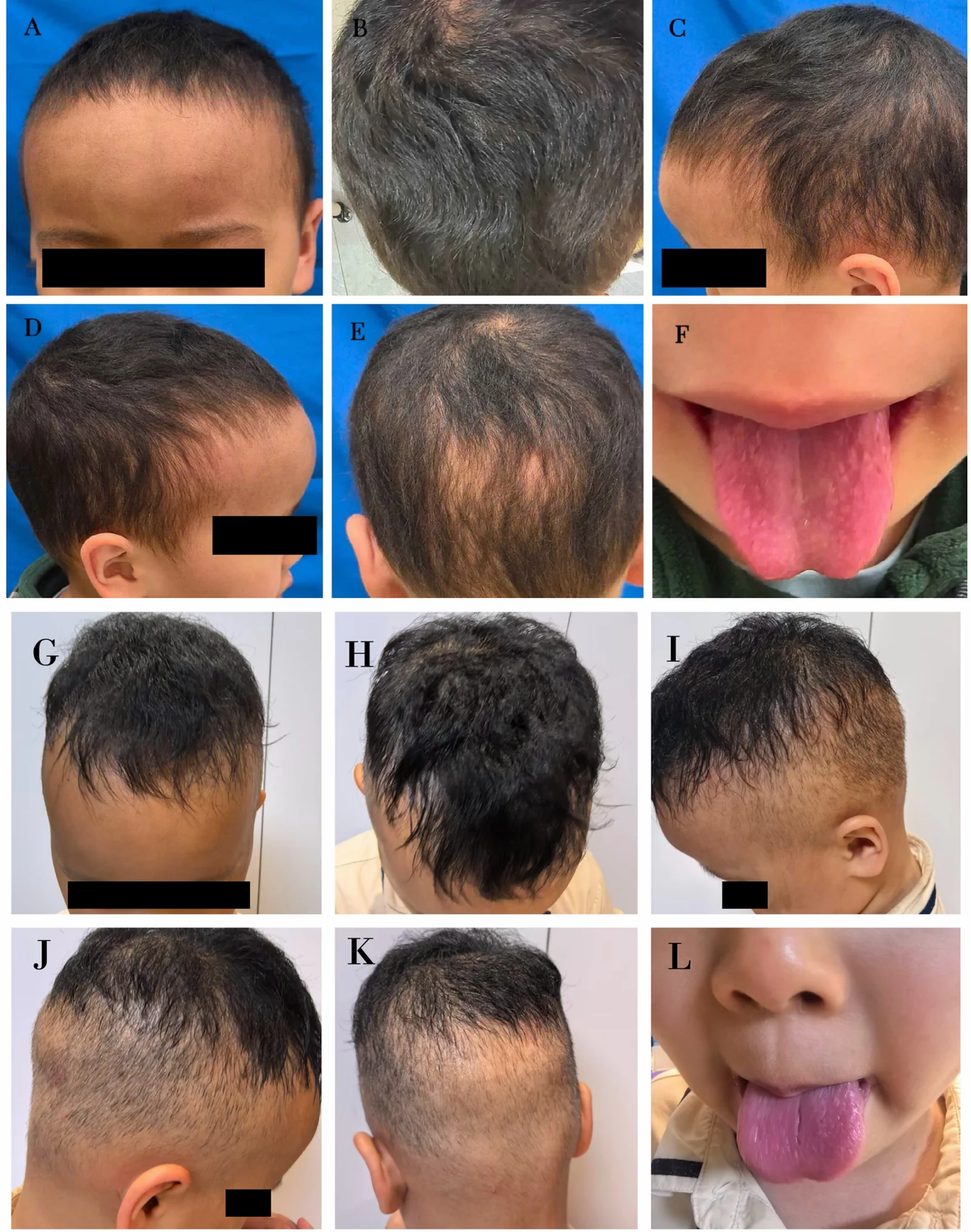
Clinical appearance at the 3–month and 5–month follow–ups. **(A–F)** Clinical appearance at the 3–month follow–up, showing visible hair regrowth across the frontal, parietal, temporal, and occipital scalp **(A–E)** and the oral mucosa **(F)**. **(G–L)** Clinical appearance at the 5–month follow–up. Frontal hair density remained relatively stable **(G)**, whereas recurrent hair loss was evident in the bilateral temporal regions **(I, J)** and occipital scalp **(K)**. The oral mucosa is shown in panel L.

**Figure 3:**
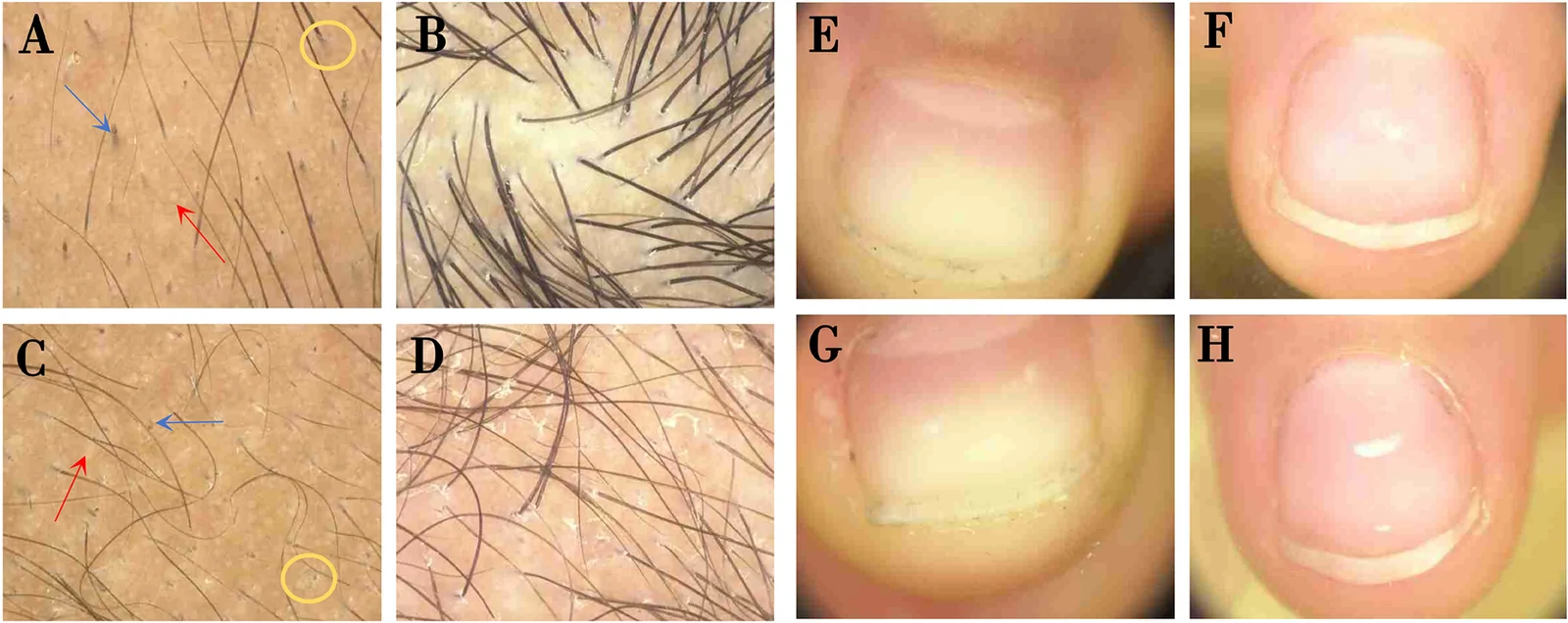
Trichoscopic and nail findings at baseline and after 3 months of treatment. **(A,C)** Baseline trichoscopy of the vertex **(A)** and whorl **(C)** regions showing diffuse hair thinning, vellus hairs, yellow dots (red arrows), black dots and broken hairs (blue arrows), and perifollicular keratosis (yellow circles). **(B,D)** Trichoscopy of the corresponding regions at the 3-month follow-up showing fewer yellow dots, increased vellus and terminal hairs, and reduced perifollicular keratosis. **(E,G)** Baseline examination of both thumbnails showing mild nail pitting (white arrows). **(F,H)** Three-month follow-up showing improvement in nail pitting. Imaging parameters: A–D, Hongxin dermoscope, polarized light, ×40; E–H, Hongxin dermoscope, polarized light, ×20.

At the approximately 8-month follow-up on 27 August 2026, the mother of the child, then aged 6 years and 1 month, further reported that another episode of hair loss had occurred after a febrile illness in July. The occipital scalp was again most prominently affected, followed by gradual regrowth beginning in early August. Topical scalp treatment was continued throughout this period. This course suggested a fluctuating pattern of hair involvement, with a temporal association between worsening hair loss and febrile episodes. No new recurrence of ocular inflammation was documented during follow-up, and the patient remains under ongoing follow-up. At the latest parental history update during manuscript revision, the halcinonide–minoxidil mixture and hydroxychloroquine remained in use. The parents reported good adherence and no adverse events; these assessments were based on parental report rather than standardized adherence measures or systematic safety testing.

## 5 Discussion

A major feature of this family is the marked phenotypic variability associated with the same *SREBF1* variant. Both the patient and his father carried *SREBF1* NM_004176.5:c.1580G>A (p.Arg527His), which corresponds to NM_001005291.2:c.1670G>A (p.Arg557His), previously reported in HMD (4). Their clinical presentations differed substantially. The child developed recurrent, extensive non-scarring alopecia from 18 months of age, together with severe ocular surface disease, corneal ulceration, recurrent oral ulcers, and nail pitting. His father developed alopecia at approximately 8–9 years of age and currently has only mild occipital and temporal hair loss, recurrent conjunctival hyperemia, and dry eye, with no known history of corneal ulceration or oral involvement. The elder sister, who did not carry the variant, was clinically unaffected. Variable expression among affected members of *SREBF1*-related families has been described previously (3, 4). In this family, the differences involved age at onset, extent and severity of hair loss, and the degree of ocular and mucosal involvement. Mildly affected adult carriers may therefore present only with non-specific alopecia or minor ocular surface symptoms and may be overlooked during routine family history taking. The father's ocular history was self-reported, and the absence of a formal ophthalmologic assessment limits comparison of ocular severity between father and son.

Another feature was the recurrent fluctuation of hair loss in temporal association with febrile episodes. On several occasions, marked shedding occurred on the day of fever or within several days to 1–2 weeks after defervescence, with the occipital scalp most affected, followed by gradual regrowth over the subsequent weeks. The same pattern was reported retrospectively by the family and was observed again during follow-up in 2026. Cyclic or recurrent alopecia has been described in other patients with *SREBF1*-related disease. Kim et al. reported two infants with *SREBF1*-associated HMD whose scalp hair loss followed a cyclical pattern (5). Park et al. described a child with intermittent, diffuse scalp hair shedding followed by partial regrowth, without clear periodicity or an identifiable external trigger (6). This case documents recurrent alopecia with a repeatedly reported temporal association with febrile illness and predominant occipital involvement.

High fever and systemic illness are recognized triggers of telogen effluvium (7). However, acute telogen effluvium typically becomes apparent several weeks to months after the triggering event, commonly around 2–3 months later (7). The much shorter interval observed in our patient—from the day of fever to several days or 1–2 weeks after defervescence—is therefore not typical of conventional telogen effluvium. The present observations do not establish a specific causal relationship between fever and *SREBF1*-related hair loss. They document a repeatedly observed temporal pattern that warrants comparison with future cases and may help define the natural history of recurrent alopecia in this disorder.

The phenotype also fits the evolving view that *SREBF1*-associated HMD and the condition previously described as autosomal dominant IFAP (AD-IFAP) belong to the same clinical spectrum. Wang et al. first identified heterozygous *SREBF1* variants in patients described as having AD-IFAP (1). Irurzun et al. subsequently described substantial clinical overlap between IFAP and HMD (2), and Murase et al. specifically proposed that *SREBF1*-associated HMD and AD-IFAP represent a clinical spectrum caused by *SREBF1* variants (8). More recently, Slater et al. applied ClinGen lumping and splitting criteria and concluded that the two phenotypes are better regarded as variable expression of the same *SREBF1*-related disorder (3). Our patient had non-scarring alopecia, follicular papules, and perifollicular keratosis, features described within the AD-IFAP phenotype, together with oral mucosal, nail, and severe ocular surface involvement, but without prominent photophobia. We therefore consider *SREBF1*-associated HMD the most appropriate diagnosis and interpret the phenotype within the HMD–AD-IFAP spectrum. This family does not independently establish that the two previously named conditions are a single disease entity, but its segregation pattern and marked variable expression are consistent with the proposed unified spectrum.

Ocular involvement in this patient is also consistent with the reported spectrum of *SREBF1*-associated disease. Kim et al. described recurrent vascularizing keratitis with stromal opacity in infants with *SREBF1*-associated HMD (5). Long-term ophthalmologic follow-up by Feizi et al. documented meibomian gland dysfunction, superficial corneal vascularization, and corneal opacity (9). In addition, Butovich et al. found an altered meibum lipid profile in a patient carrying an *SREBF1* p.Arg527Cys variant, including an increased ratio of saturated to unsaturated wax esters, and proposed a possible link between abnormal meibum composition and meibomian gland dysfunction (10). Our patient had obstruction of the meibomian gland orifices, lipid plugging, BKC, dry eye, and corneal ulceration. These findings fall within the reported ocular phenotype, although the lipidomic observations from another *SREBF1* variant should not be assumed to establish the mechanism of ocular disease in the present patient.

The recognized phenotype has also expanded beyond the skin, hair, mucosa, and eyes. Slater et al. recently reported gastrointestinal involvement in *SREBF1*-related disease, including gastroesophageal reflux disease and esophageal strictures or webs (3). Neither the patient nor his father had dysphagia, food impaction, recurrent vomiting, or other significant gastrointestinal symptoms. The combination of affected and unaffected organ systems in this family is consistent with the broad and variably expressed phenotype of *SREBF1*-related disease. Gastrointestinal assessment was based on reported symptoms; the absence of symptoms does not exclude asymptomatic structural abnormalities.

The diagnostic course also has practical relevance. Early in the disease, recurrent alopecia, ocular inflammation, and abnormal autoantibody results led to evaluation for alopecia areata, immune-mediated alopecia, and connective tissue disease, followed by treatment with hydroxychloroquine and baricitinib. In a child with recurrent non-scarring alopecia, concurrent ocular surface disease, follicular keratinization, oral or nail abnormalities, and a positive family history should raise suspicion of an inherited epithelial disorder. Autoantibody positivity alone may not account for such a multisystem phenotype. Molecular diagnosis can place the hair, ocular, mucosal, and nail findings within a single disease framework and may reduce prolonged use of immune-directed treatments when a clear autoimmune indication is lacking.

Interpretation is limited by the single-family design, the partly retrospective and parent-reported timing of febrile episodes and hair loss, and the naturally fluctuating course. Hair regrowth during concurrent topical and systemic treatment cannot be attributed to a specific intervention. These observations therefore do not establish treatment efficacy or a causal effect of fever on alopecia.

## 6 Conclusion

The main findings in this family are the marked intrafamilial variability associated with the same *SREBF1* variant and the longitudinally documented pattern of recurrent hair loss temporally associated with febrile episodes. The phenotype is also consistent with the proposed HMD–AD-IFAP spectrum. Early genetic evaluation should be considered when childhood alopecia occurs together with ocular and other epithelial manifestations.

## 7 Patient perspective

The patient's family expressed overall satisfaction with the clinical management and the partial improvement achieved with topical therapies. They noted with encouragement that hair regrowth in the frontal region was maintained, though they acknowledged the recurrent shedding in the occipital area.

## Data Availability

The original data supporting the findings of this study are available from the corresponding author upon reasonable request.
