# Supplementary material for: Case Report: Marked intrafamilial variability and recurrent fever-associated alopecia in SREBF1-associated hereditary mucoepithelial dysplasia
Source: Front Med (Lausanne). 2026 Oct 1;13:1910789. doi: 10.3389/fmed.2026.1910789 (PMC13668859; doi:10.3389/fmed.2026.1910789)
Supplement: Supplementary file 2 [file Datasheet2.pdf]

Completed CARE checklist

Manuscript 1910789 | Frontiers in Medicine

Case Report: Marked Intrafamilial Variability and Recurrent Fever-Associated Alopecia in SREBF1-Associated Hereditary Mucoepithelial Dysplasia

Item numbering follows the 2013 CARE checklist. Descriptions below are abbreviated. Locations refer to the accompanying 10-page revised manuscript. Unavailable details are explicitly identified rather than marked as completed evidence.

Item	Reporting topic	Location	Completion and explanation
1	Title	p. 1	Diagnosis and case-report designation included.
2	Keywords	p. 1	Five keywords, including case report.
3a	Abstract contribution	p. 1	Family variability and fever-associated recurrence summarized.
3b	Abstract presentation	p. 1	Alopecia, ocular disease, oral ulcers and nail findings described.
3c	Abstract management and outcome	p. 1	Molecular diagnosis, topical treatment and recurrent shedding reported.
3d	Abstract lesson	p. 1	Clinical contribution stated without a causal claim.
4	Introduction	p. 1, section 1	Clinical context and reason for reporting this family.
5a	Patient description	pp. 1–2, section 2	Age and sex reported; direct identifiers excluded.
5b	Presenting concerns	pp. 1–2, section 2	Hair loss, fever-related history and associated manifestations.
5c	Personal and family history	p. 2, section 2; pp. 2–3, section 3	Relevant family and genetic history reported. A detailed psychosocial history was not available.
5d	Previous treatment	p. 2, section 2; p. 7, Table 1	Minoxidil, hydroxychloroquine and prior baricitinib described; no appreciable improvement during baricitinib reported by parents.
6	Examination findings	p. 2, sections 2–3; pp. 8, 10	Clinical, ophthalmologic, trichoscopic and nail findings.
7	Timeline	p. 7, Table 1	Onset, diagnostic workup, treatment and dated follow-up.
8a	Diagnostic methods	pp. 2–3, section 3	Laboratory, imaging, trichoscopy, ophthalmology and genetic testing.
8b	Diagnostic difficulties	p. 2, sections 2–3; pp. 4–5, section 5	Autoantibody findings and initial immune-mediated differential complicated recognition.
8c	Diagnosis and alternatives	pp. 2–3, section 3; pp. 4–5, section 5	HMD favored; alopecia areata, connective tissue disease and telogen effluvium discussed.
8d	Prognostic assessment	pp. 3–5, sections 4–5	No formal prognostic staging applied; fluctuating course and limitations described.
9a	Treatment types	p. 3, section 4; p. 7	Systemic, topical and ophthalmologic management described.
9b	Dose and duration	pp. 2–3, sections 2 and 4; p. 7	Mixture: 1 mL of each stock solution, 2 mL twice daily; ongoing use. Hydroxychloroquine 75 mg/day in treatment record. Baricitinib about 6 months; dose and exact dates unavailable. Detailed schedules of additional topical/ocular treatments unavailable.
9c	Treatment changes	pp. 2–3, sections 2 and 4; p. 7	Baricitinib stopped before January 2026 and not restarted; ongoing hydroxychloroquine and topical mixture. Exact stopping date/reason was not established beyond parent-reported lack of appreciable improvement.
10a	Follow-up outcomes	p. 3, section 4; p. 7; pp. 9–10	Clinical regrowth and recurrent occipital shedding; parental updates distinguished from examinations.
10b	Follow-up assessments	p. 3; pp. 9–10	Serial clinical photographs, trichoscopy and nail findings.
10c	Adherence and tolerability	p. 3, section 4; p. 7	Good adherence and tolerability reported by parents; no standardized measure used.
10d	Adverse events	p. 3, section 4; p. 7	No adverse events reported by parents; systematic safety testing not documented.

Item	Reporting topic	Location	Completion and explanation
11a	Strengths and limitations	pp. 3–5, section 5	Family comparison and longitudinal course; single-family, retrospective and combined-treatment limitations.
11b	Literature context	pp. 3–5, section 5	Relevant molecular, phenotypic and ocular literature discussed.
11c	Interpretation	pp. 3–5, section 5	Genotype–phenotype interpretation and cautious fever association.
11d	Concluding lesson	p. 5, section 6	One-paragraph conclusion without references.
12	Family perspective	p. 5, section 7	Caregiver perspective presented for the young child.
13	Publication consent	p. 5, Ethics statement	Guardian consent for the child and images, and separate paternal consent for his information reported; signed forms retained by authors.

Source: CARE Case Report Guidelines, 2013 checklist. https://www.care-statement.org/checklist (accessed September 2026).
